# Factors Influencing Patient-Centered Care and Shared Decision-Making in Primary Care Settings in Saudi Arabia: A Scoping Review

**DOI:** 10.7759/cureus.76142

**Published:** 2024-12-21

**Authors:** Monzir A Alfattani, Wed A Bakudam, Waleed I Alharbi

**Affiliations:** 1 Family Medicine, Albarzah Primary Health Care Center, Makkah Health Cluster, Ministry of Health, Makkah, SAU; 2 Surgical Affairs, Royal College of Surgeons in Ireland, Dublin, IRL; 3 Medicine, University College Dublin, Dublin, IRL

**Keywords:** family medicine, patient-centered care, primary care, saudi arabia, shared decision-making

## Abstract

The Kingdom of Saudi Arabia has undergone many reforms in light of Vision 2030, and the health sector is no exception. Vision 2030 highlights the importance of utilization and increased quality in primary care. Patient-centered care (PCC) and shared decision-making (SDM) are two methods used to increase healthcare quality globally. However, there is limited research on the implementation of such methods in primary care within Saudi Arabia. Thus, this scoping review aims to address this gap by investigating the different factors influencing the implementation of PCC and SDM in primary care in Saudi Arabia. The scoping review completed a comprehensive search of three databases, Scopus, PubMed, and Web of Science, resulting in eight articles that met the inclusion criteria. The review found three major themes of influencing factors: patient-specific factors, physician-specific factors, and health system and external factors. The review highlights the lack of research in this area and provides context for the implementation of new reforms and policies in relation to PCC and SDM in primary care in the Kingdom of Saudi Arabia. Most notably, it emphasizes the importance of increasing health literacy, improving physician training, investigating cultural influence, and enhancing policies.

## Introduction and background

The healthcare system is founded on the principles of quality and accessibility, with patient-centered care (PCC) and shared decision-making (SDM) increasingly recognized as significant contributors in recent years [1]. PCC emphasizes prioritizing the patients' values, needs, and wants, understanding patients’ conditions from their own perspectives, and putting them in the context of their own unique lives [2]. Similarly, SDM promotes the inclusion of the patient as well as other healthcare professionals in making decisions related to the patient’s treatment and healthcare, broadening the perspective of the patient’s condition in the wider context of the individual’s life and other circumstances [3]. Thus, PCC and SDM are interrelated, proving to complement one another in the pursuit of increased patient satisfaction and quality of healthcare [4].

Primary care is the foundational level of healthcare delivery, proven to be the basis of providing high-quality healthcare [5]. Therefore, it is important to advance the quality of primary care to advance quality in overall healthcare, highlighting the importance of implementing PCC and SDM in primary care. Since the implementation of Vision 2030, the Kingdom of Saudi Arabia has made a plethora of reforms in the health system. Through the National Transformation Plan and the implementation of the new model of care, the Saudi health system has seen improvements, including increased participation of the private sector, digitalization of health through digital health apps, expansion of the workforce by opening more physician and training positions, enhanced healthcare financing through the establishment of a value-based system, corporatization of hospitals and health institutions through the creation of health networks, and improved governance of health systems through the introduction of “super-regulators” [6]. A significant aim of Vision 2030 in Saudi Arabia is to enhance primary care across the country by effectively utilizing primary care centers and improving their quality. Furthermore, the vision for the country’s healthcare system emphasizes increased involvement of the patient’s perspective in reaching experts’ decisions regarding treatment and care [6]. Thus, it is clear that PCC and SDM are important pillars in achieving the aims of the Kingdom of Saudi Arabia in primary care and overall healthcare.

While the global literature underscores the importance and scope of PCC and SDM implementation in primary care, it is a new and understudied topic in the context of Saudi Arabia [7]. The unique cultural context, ongoing healthcare reforms, and systemic factors create specific facilitators and barriers that require investigation to support effective integration.

This scoping review aims to address this gap by further understanding the different factors influencing the implementation of PCC and SDM in primary care in Saudi Arabia. Through synthesizing findings from relevant literature, this review seeks to provide insight into the current state of PCC and SDM in primary care, identify key challenges and facilitators, and propose areas for future research.

## Review

Methodology

Study Design

This scoping review was conducted following Arksey and O’Malley’s methodological framework, supplemented by the Preferred Reporting Items for Systematic reviews and Meta-Analyses (PRISMA) extension for Scoping Reviews checklist for reporting findings [8,9].

Research Questions

1) What factors influence PCC and SDM in primary care in Saudi Arabia?

2) In what ways and to what extent are PCC and SDM factors in primary care in Saudi Arabia interrelated?

Search Strategy

A comprehensive search was performed across three databases, Scopus, Web of Science, and PubMed, between November 2024 and December 2024 by reviewers (M.A.A. and W.A.B.). A combination of the following search strings and terms was used: ("patient-centered care" OR "person-centered care" OR "shared decision making" OR "SDM" OR "patient involvement" OR "collaborative care") AND ("primary care" OR "family medicine" OR "general practice" OR "primary healthcare" OR "community health" OR "general practitioners") AND ("Saudi Arabia" OR "KSA" OR "Kingdom of Saudi Arabia" OR "Saudi").

Article Selection and Eligibility Criteria

Articles were searched and screened in a two-step process by two reviewers (M.A.A. and W.A.B.). First, titles and abstracts were screened by the two reviewers separately for adherence to topic relevance. Discrepancies between the reviewers were resolved through discussion. Subsequently, the same two reviewers separately screened included full-text studies for alignment with inclusion criteria, and similarly, upon completion, reviewers compared results, and discrepancies were resolved through discussion. Articles were selected based on the following inclusion criteria: focusing on primary care settings in Saudi Arabia, addressing PCC or SDM, being available in English, and providing sufficient methodological details for quality assessment. No restrictions were placed on study design, year of publication, or article type.

Quality Assessment

The two reviewers (M.A.A. and W.A.B.) individually appraised articles that met the inclusion criteria using the 11 quality indicators for selection developed by Buckley et al. [10]. Discrepancies between the two reviewers were resolved through discussion. Only articles deemed of high quality were included; any article that showed bias and did not meet the quality standard was excluded.

Data Charting and Analysis

Data from included studies were charted into a standardized table (see Table 1), extracting variables of study authorship, year of publication, institutional affiliations, study aim, and study design. Analysis of relevant variables was conducted, and a thematic analysis of the eight study findings was conducted to identify patterns of PCC and SDM-related factors, addressing the research questions.

**Table 1:** Articles’ general information SDM: shared decision-making

Study	Year of publication	Title	Journal	Institution	Paper aim	Study design
AlHaqwi et al. [11]	2015	Shared clinical decision making: a Saudi Arabian perspective	Saudi Medical Journal	King Saud bin Abdulaziz University for Health Sciences	To determine the preferences of patients regarding their involvement in the clinical decision-making process and the related factors in Saudi Arabia	Cross-sectional study
Alsulamy et al. [12]	2022	Healthcare professionals' views on factors influencing shared decision-making in primary health care centres in Saudi Arabia: a qualitative study	Journal of Evaluation in Clinical Practice	The University of Sheffield	To describe the perspectives of healthcare professionals regarding the implementation of SDM in primary healthcare centers in Saudi Arabia	Qualitative semistructured interviews
AlHaqwi et al. [13]	2023	Impact of patient-centered and self-care education on diabetes control in a family practice setting in Saudi Arabia	International Journal of Environmental Research and Public Health	King Saud bin Abdulaziz University for Health Sciences	To assess the impact of patient-centered diabetes education sessions on the prescribed treatment plan in controlling diabetes and other related cardiovascular risk factors	Preexperimental pretest-posttest one group study
Alhalal et al. [14]	2023	The effect of health literacy on health-related quality of life among Saudi Women with chronic diseases	The Journal of Nursing Research	King Saud University	This study was designed to examine the relationship between health literacy and health-related quality of life and determine the mediating roles of healthy lifestyle and patient-centered care in explaining this relationship	Cross-sectional study
Alomran and Alyousefi [15]	2023	Attitudes of family medicine trainees towards patient-centeredness practice	International Journal of General Medicine	King Saud University	This study explores family medicine residents’ attitudes toward the physician-patient relationship and patient-centered care and the possible influence of demographic characteristics, level of training, school of graduation, and previous training	Cross-sectional study
Amin et al. [16]	2020	Does shared decision making increase prostate screening uptake in countries with a low prevalence of prostate cancer?	African Health Sciences	King Saud University	To investigate whether SDM increases the uptake of prostate cancer screening practices among Saudi men	Community-based study
Alrawiai [17]	2023	Deprescribing, shared decision-making, and older people: perspectives in primary care	Journal of Pharmaceutical Policy and Practice	Imam Abdulrahman Bin Faisal University	This review aims to examine the current situation of deprescribing, especially in primary care settings, and how SDM can be used to optimize the deprescribing process	Review
Leila A Boubshait [18]	2022	Patient trust in primary care physicians: a mixed methods study of persons with diabetes at university- based clinics in the Eastern province of Saudi Arabia	Patient Preference and Adherence	Imam Abdulrahman Bin Faisal University	This study aims to explore patients’ perceptions of trust in physicians, determine factors that play a role in this relationship, and identify ways to improve patient trust	Mixed methods

Results

PRISMA Flow Diagram

**Figure 1:**
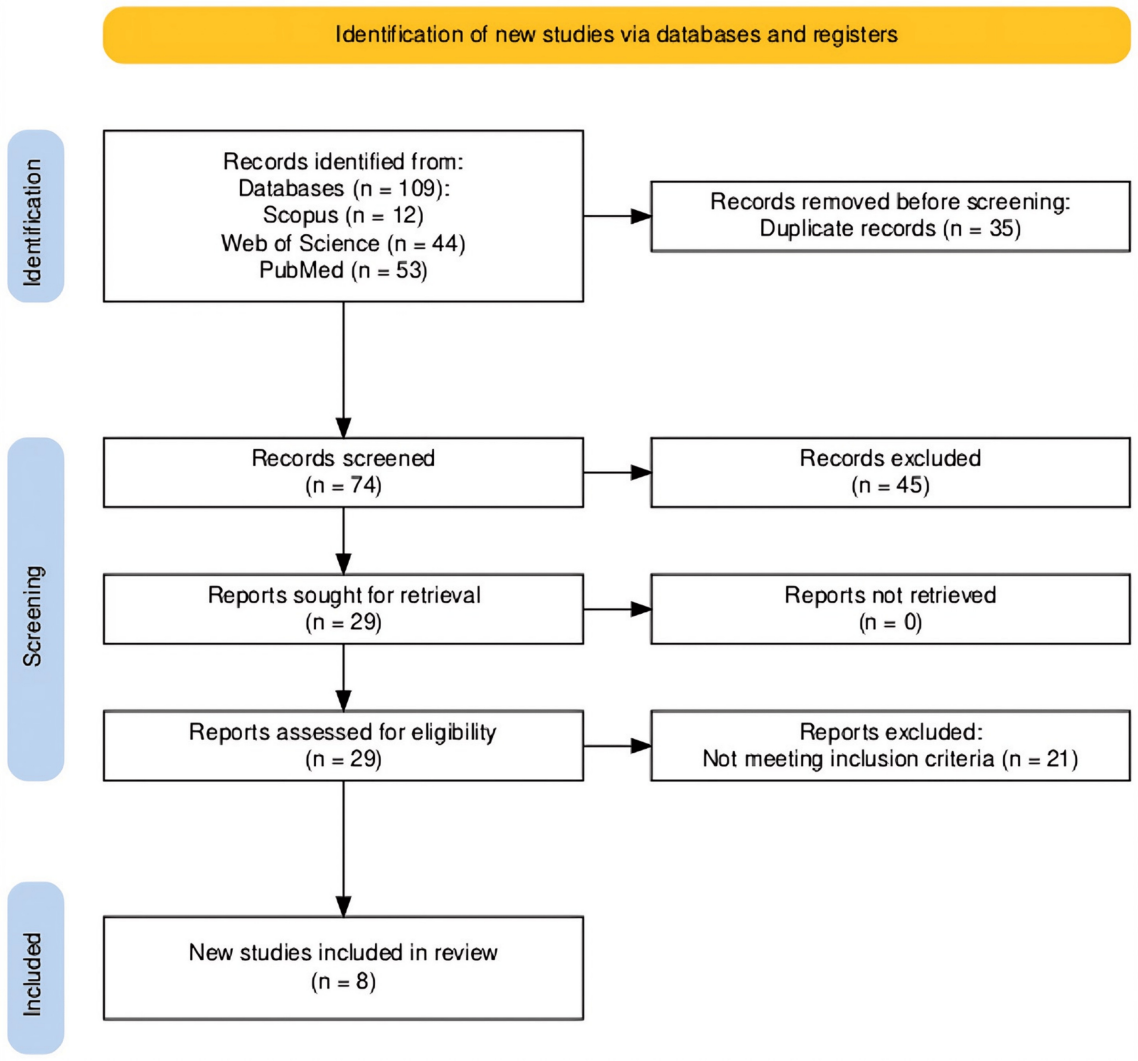
PRISMA flow diagram Source: [19]

The results of the literature search are summarized in Figure 1 using the PRISMA flow diagram template [19]. The initial search identified 109 articles, of which 35 were removed as duplicates. Of the remaining 74 articles, their titles and abstracts were screened, and 29 qualified for full-article retrieval. All selected articles were retrieved and screened for alignment with the inclusion criteria, of which only eight articles were identified for the final selection and adherence with the research questions and inclusion criteria.

According to Authorship

As shown in Table 2, the eight studies were authored by nine contributors, with one article featuring dual primary authorship. Two articles were authored by Ali I. AlHaqwi (22.22%), while the remaining articles were authored individually by other researchers.

**Table 2:** Main authorship

Main author	Frequency (n = 9)	Percentage
Ali I. AlHaqwi [13]	2	22.22
Sumaiah Alrawiai [17]	1	11.11
Amal Alomran [15]	1	11.11
Nada Alyousefi [15]	1	11.11
Eman Alhalal [14]	1	11.11
Nouf Alsulamy [14]	1	11.11
Leila A. Boubshait [18]	1	11.11
Hussein Saad Amin [13]	1	11.11

According to Year of Publication

**Figure 2:**
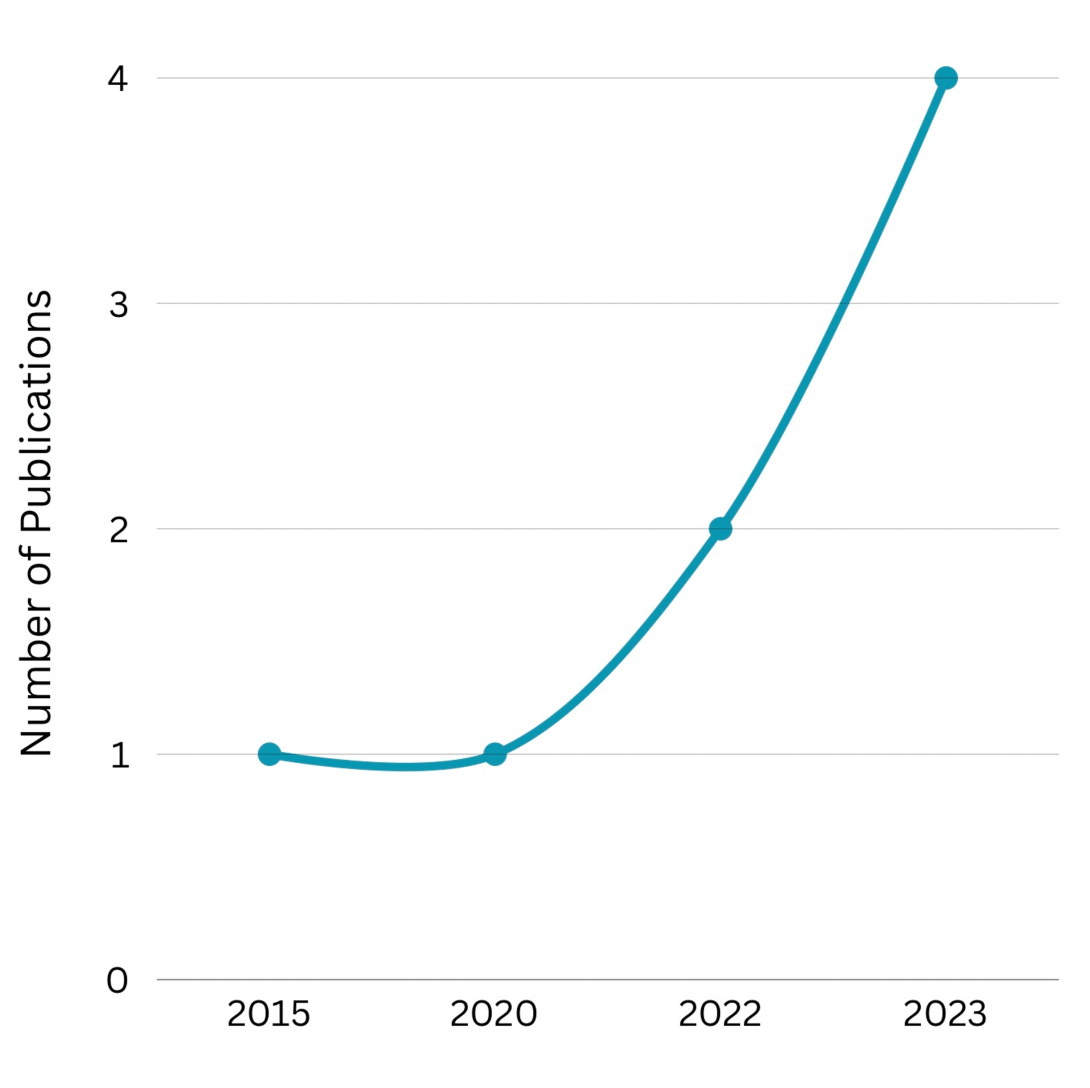
Frequency of included articles’ publication year Source: This is an original image created by the author Wed A. Bakudam.

Figure 2 showcases the trend in the year of publication of the eight selected articles. Of these, half of the articles were published in 2023 (50%), followed by two articles being published in 2022 (25%), and one article being published in both 2020 (12.5%) and 2015 (12.5%), respectively. This indicates a recent interest in the topic of PCC and SDM in primary care in Saudi Arabia, with 75% of the articles included being from the past three years.

According to Institutional Affiliation

As shown in Table 3, four institutions contributed to the included studies. Three articles were affiliated with King Saud University (37.5%), while both Imam Abdulrahman Bin Faisal University and King Saud bin Abdulaziz University for Health Sciences produced two articles each (25%). Finally, one article was contributed by the University of Sheffield (12.5%).

**Table 3:** Institutional affiliation

Institutions	Frequency (n = 8)	Percentage
King Saud University	3	37.5
Imam Abdulrahman Bin Faisal University	2	25
King Saud bin Abdulaziz University for Health Sciences	2	25
The University of Sheffield	1	12.5

According to Study Design

Table 4 showcases a diverse range of study designs included. Three of the eight articles selected were cross-sectional studies (37.5%), with the rest being of different study designs. Overall, the included articles were a mix of cross-sectional studies, review articles, qualitative semistructured interviews, mixed methods, preexperimental pretest-posttest one-group studies, and community-based studies.

**Table 4:** Study design

Study design	Frequency (n = 8)	Percentage
Cross-sectional study	3	37.5
Review	1	12.5
Qualitative semistructured interviews	1	12.5
Mixed methods	1	12.5
Preexperimental pretest-posttest one group study	1	12.5
Community-based study	1	12.5

According to Paper Aim

The aims of the different studies included were analyzed, and three main categories were concluded. As shown in Table 5, of the eight articles selected, four were patient-based (50%), relaying the perspectives and impacts of patients. After this, two were physician-based (25%), similarly relaying the perspectives of physicians. Finally, the remaining two articles were mixed (25%), covering the topic from both the patients’ and physicians’ perspectives. Thus, it is important to note that PCC and SDM in primary care in Saudi Arabia are often analyzed and studied from patients’ perspectives, leaving a gap in physician inputs in the literature.

**Table 5:** Paper aims

Paper aim	Frequency (n = 8)	Percentage
Patient-based	4	50
Physician-based	2	25
Mixed	2	25

Study Finding Thematic Analysis

**Figure 3:**
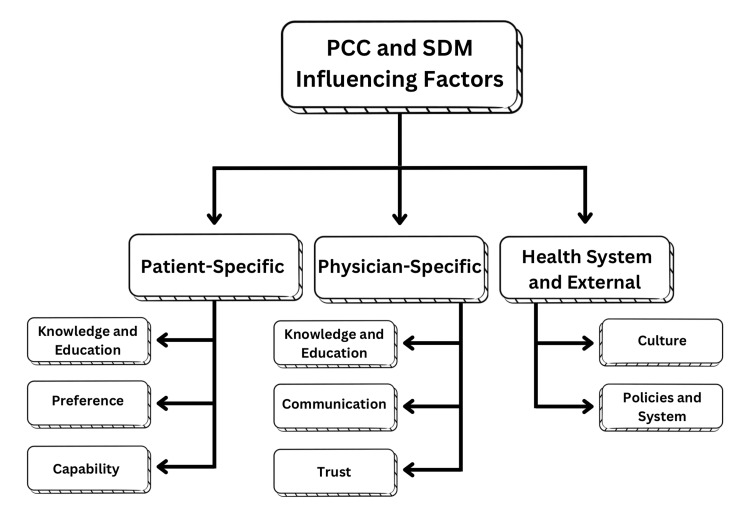
PCC and SDM influencing factors thematic analysis map PCC: patient-centered care; SDM: shared decision-making Source: This is an original image created by the author Wed A. Bakudam.

The included studies were thematically analyzed and presented in Figure 3. Three main themes were identified: patient-specific factors, physician-specific factors, and health system and external factors.

Patient-Specific Factors

Multiple articles discussed the influence of patient factors on implementing PCC and SDM in primary care [11-14]. Articles revealed that knowledge and level of education influenced the extent to which an individual was able to contribute to the decision-making of their health plan [11-14]. Furthermore, multiple factors, including age, gender, and level of education, influenced the preferences of patients regarding their approach to their own healthcare. A study led by AlHaqwi et al. revealed a direct correlation influencing individuals’ preferences on SDM, as the preference for being collaboratively involved in SDM was higher among males and individuals with a more advanced level of education [11]. Finally, articles acknowledged that patients' capability posed a prominent factor, as individuals with certain conditions or health statuses are largely unable to successfully participate in their own healthcare due to health limitations. For instance, as illustrated by Alsulamy et al., it is easier for individuals with only one condition, such as diabetes, to understand the treatment options and risks involved. However, individuals with more than one condition, such as diabetes, hypertension, and heart conditions, find it more difficult due to the compounded effects of treatment options and associated risks [12].

Physician-Specific Factors

Similar to patient-specific factors, multiple articles highlighted physicians’ knowledge and education as contributing factors to implementing PCC and SDM in primary care [12,15-18]. However, regarding physicians, articles highlighted their knowledge and training about the steps of successfully implementing PCC and SDM. Multiple articles suggested the introduction of PCC and SDM training courses, as well as integration in medical curricula, as an approach to increase physicians’ awareness and capability in its integration in patient care [12,15,16]. Furthermore, one of the most significant factors highlighted throughout is physicians’ communication ability, as well as the trust built between patients and physicians, being the basis for successfully implementing patient care [12,17,18].

Health System and External Factors

Aside from both physicians and patients, some articles highlighted the effect of the health system itself, as well as external factors, on the implementation capacity of PCC and SDM [11,12,18]. Cultural context was highlighted as an important point of investigation in relation to its effects on patients’ preferences for contributing to their healthcare plans. A study based in Saudi Arabia compared the preference of females leaning more passive than the existing literature evidence of females in Western cultures [11]. Furthermore, health system policies are factors driving PCC and SDM integration; gaps in policies and guidelines that do not support the implementation of such approaches are a crucial point of investigation and improvement. For instance, the importance of physician continuity in a patient’s care and follow-up allows physicians to take patient care in a holistic manner, considering the patient’s context rather than simply treating their illnesses [11,18]. In addition, the healthcare system’s impact on the amount of work pressure on physicians directly affects physicians’ capabilities to provide adequate time in patients’ consultations to implement PCC and SDM [12].

Discussion

To the best of our knowledge, the topic of PCC and SDM has not been studied in a review article in the context of primary care in Saudi Arabia. The number of studies included, eight from a total initial search of 74, showcases the lack of literature on PCC and SDM in the context of primary care in Saudi Arabia. This is further reinstated by the results of the included studies’ year of publication, which was heavily saturated in the last three years with a steep upward trend. Furthermore, the eight articles being affiliated with only four institutions showcases that while interest is on the rise, it is still not as widespread, being concentrated within specific institutions. The results of the study showcase that the topic of PCC and SDM in primary care in Saudi Arabia lacks the integration of the physician perspective. However, PCC and SDM are both methods of active collaboration between physicians and patients. Thus, it is more beneficial to direct future research to integrate both perspectives in synthesizing results [20].

The study identified three themes, one of which was patient-specific factors influencing the implementation of PCC and SDM, of which health literacy levels and patients' educational background were significant contributors to the extent of implementing the methods. A study led by Roodbeen et al. investigated the barriers and facilitators of SDM in patients with limited health literacy, citing its significant potential effect on SDM implementation, as well as the recommendation of extended consultation time and enhanced communication to combat the barriers resulting from limited health literacy in patients [21]. Furthermore, the subtheme of patients' preference was an apparent outcome, as patients cannot be forced to engage actively in decisions about their health plans if they prefer the passive role. However, it is important to note here that knowing and understanding the patient's preference is within itself adherence to personalized care and PCC [22]. Second, the study identified physician-specific factors, and communication is underscored as one of the leading factors influencing the implementation and extent of PCC and SDM from both the patient and physician perspectives. Similarly, the studies have also highlighted that trust between physicians and patients plays a role in the extent to which patients are willing to engage with physicians. More notably, it is the interplay between communication and trust that creates the basis for successful PCC and SDM implementation. The better the communication skills of the physician, the higher the level of patient-physician communication and the more trust a patient feels in his health professional. Similarly, the more trust is built between patients and physicians, the more information a patient is comfortable sharing with his caregiver, and the better patient-physician communication becomes. Thus, both communication and trust complement each other to expand the scope of PCC and SDM implementation. Furthermore, the studies highlighted the importance of physicians’ training and knowledge in implementing the methods. A systematic review led by Waddell et al. highlighted that the lack of trust in physicians' own ability and the underdevelopment of physicians’ communication skills were leading barriers to the implementation of SDM [23]. Patients and physicians are the center factors of both PCC and SDM; thus, it is advantageous to know the factors related to them. However, they do not exist in a vacuum, and thus, health systems and external factors were identified as a separate theme. The cultural, religious, and personal beliefs of individuals greatly influence their interaction with the health system and primary care; therefore, it is important for physicians to develop cultural competence and understand the overall impact of these factors on their patients' decisions [24]. Studies included highlight the need to further explore the cultural context of Saudi Arabia and its impact on practicing PCC and SDM. A study led by Sheeran et al. investigated the cultural influence on the implementation of PCC, which found similarities in different cultures in the values and importance of SDM, empathy, and information exchange. However, the study found differences between cultures in how that information is shared [25]. Furthermore, aligning with this study’s results, a publication by Yahanda and Mozersky highlighted time constraints as a factor holding back the successful implementation of SDM and PCC [26]. Health system policies regulate the extent to which physicians can implement the methods, as high work pressure and shorter consultations, with a lack of a properly implemented documentation system, can negatively affect the physician’s understanding of the patient context and thus decrease PCC and SDM [27]. Thus, it is the intercalation of the patient, physician, health system, and external factors that contribute to the implementation of PCC and SDM.

Recommendations and implications for practice

Analyzing the included articles, multiple recommendations and improvements were synthesized for developing PCC and SDM implementation in primary care in Saudi Arabia. First, enhancing patients' health literacy and empowerment will increase the extent to which patients will engage in PCC and SDM. Health literacy education programs, such as seminars, creating easy-to-read pamphlets, providing platforms for patient advocates, and appointing patient ambassadors, can be tangible ways to make progress toward this objective. The introduction of PCC and SDM training for physicians and healthcare professionals will enhance their capacity to implement these methods. Integrating communication training into medical school curricula and providing simulation training to physicians on key steps to successfully involve patients in decision-making, as well as equipping them with sufficient information about the risks and benefits of treatments, are essential for enabling patients to make informed decisions about their healthcare plans. Third, this study highlights and acknowledges the gap in the literature in investigating the cultural context of Saudi Arabia and its effect on implementing PCC and SDM in primary care. Thus, studies on such topics are recommended to fill the gap. Finally, changes in policies and reform to improve policies in continuity of care, documentation, and decreased work pressure on physicians are expected to benefit the overall implementation of PCC and SDM.

Although Saudi Arabia is currently undergoing significant reforms and system changes, it is important to approach these changes gradually. Beginning with substantial policy reform to enhance the health system, followed by integrating PCC and SDM training for physicians, and implementing these methods through active listening and effective communication will establish a strong foundation for sustainable reform. Enhancing patient literacy with cultural acknowledgment and sensitivity will facilitate the smooth implementation of these methods. Continuous monitoring and adaptive change management are crucial to promoting PCC and SDM in primary care within effective and measurable parameters.

Limitations of study

All research work is encompassed within certain limitations. Two main limitations are identified for this scoping review. First, the scoping review only screened three databases: while it is a comprehensive search, this study focuses on Saudi Arabia, and a plethora of Saudi-associated journals is not indexed within those three databases, potentially resulting in missed relevant studies. Similarly, the study limited its search to articles available in English. However, Saudi Arabia is an Arabic-speaking country, and thus, potentially relevant studies may have been missed.

## Conclusions

The study analysis and results identified patient-specific factors, physician-specific factors, and health system and external factors as three major categories of the influences of PCC and SDM implementation. The study highlights the importance of increasing number of studies and investigations on the topic, as well as recommendations for the advancement of its implementation. Most notably, the study advocates for a sequential approach, beginning with policy reform, followed by enhancing physician training and skills, promoting health literacy and patient empowerment, and ultimately addressing the need to investigate cultural influences.
